# Population-level variation shapes West Nile virus transmission potential in the *Culex pipiens* complex mosquitoes

**DOI:** 10.1371/journal.pntd.0014693

**Published:** 2026-08-28

**Authors:** Octavio Giayetto, M. Andrea Onorato, Kevin A. Rucci, Gabriel Barco, Mauricio D. Beranek, Adrián Díaz

**Affiliations:** 1 Laboratorio de Arbovirus, Instituto de Virología “Dr. J.M. Vanella” – Facultad de Ciencias Médicas, Universidad Nacional de Córdoba, Córdoba, Argentina; 2 Consejo Nacional de Investigaciones Científicas y Técnicas, Ciudad Autónoma de Buenos Aires, Buenos Aires, Argentina; Connecticut Agricultural Experiment Station, UNITED STATES OF AMERICA

## Abstract

West Nile virus (WNV; formally *Orthoflavivirus nilense*) is a globally distributed arbovirus maintained mainly by mosquitoes of the genus *Culex*. Since its introduction in Argentina in late 2004, efforts have focused on identifying which species of this genus are involved in its transmission. This study evaluated vector competence and transmission dynamics of *Culex pipiens* and *Culex quinquefasciatus* populations from central Argentina. Mosquitoes were fed using an infected bloodmeal containing increasing viral doses (from 2 log_10_ to 7.5 log_10_ PFU/mL). Infection, dissemination, and transmission were determined in Vero cells through viral plaque detection in the abdomen, legs, and saliva samples. Dose–response relationships were quantified using mixed modelling approaches incorporating population-level variation, and temporal dynamics were evaluated to estimate the extrinsic incubation period. Both species exhibited parallel dose–response patterns, indicating comparable susceptibility and transmission potential. Infection probability increased progressively with viral dose, with a median infectious dose (ID₅₀) estimated at 4.63 log₁₀ PFU/mL. Dissemination required higher viral exposure (DD₅₀ = 6.45 log₁₀ PFU/mL), while transmission showed the highest threshold (TD₅₀ = 8.16 log₁₀ PFU/mL), suggesting the presence of sequential biological barriers during the infection process. Population-level variability was most pronounced during the infection stage (14%) but became minimal once viral dissemination was established. Temporal analyses revealed that transmission began around 8 days post-infection at 27°C, with an estimated median extrinsic incubation period of 11 days. These results demonstrate that *Cx. pipiens* and *Cx. quinquefasciatus* populations exhibit comparable vector competence for WNV in Argentina and highlight infection-stage variability as a major source of heterogeneity among populations. The identified dose thresholds and incubation dynamics indicate that both species could contribute equally to local transmission cycles, providing key biological parameters for understanding WNV persistence near its southern distribution limit.

## Author summary

We studied how two common mosquito species in Argentina, *Culex pipiens* (northern house mosquito) and *Culex quinquefasciatus* (southern house mosquito) are able to transmit West Nile virus after feeding on infected blood. These mosquitoes are widely distributed and frequently feed on birds, which are the main hosts of the virus. Understanding how infection develops inside the mosquito is essential to predict when and where transmission may occur. We exposed mosquitoes to increasing amounts of virus and measured how often they became infected, how the virus spread within their bodies, and when they were able to transmit it. We found that both species respond similarly to increasing viral exposure and can transmit the virus once a threshold is exceeded. However, we also observed important differences among populations of the same species, especially during the early stages of infection. We further showed that time plays a key role: even when mosquitoes are exposed to high viral doses, transmission only occurs after several days. These findings suggest that both the amount of virus ingested and the time since infection determine transmission risk. Together, our results improve understanding of how West Nile virus may circulate in southern regions and highlight the importance of considering natural variation within mosquito populations.

## Introduction

West Nile virus (WNV; formally *Orthoflavivirus nilense*) is an arthropod-borne flavivirus recognized as one of the most widely distributed arboviruses, causing numerous documented outbreaks across Africa, Europe, Asia, Australia, and the Americas [1]. The natural transmission network of WNV is complex, although birds are the most common amplification hosts, WNV can infect a wide range of other vertebrate species, including several mammals, reptiles, and amphibians [2]. Regarding vectors, many species of mosquitoes have been involved in the maintenance network of this virus [3].

Worldwide, *Culex* species are recognized as the primary vectors of WNV [4]. Particularly, members of the *Culex pipiens* complex have been consistently identified as key WNV vectors. While *Cx. pipiens* plays a central role in sustaining viral circulation in Europe and the Middle East, *Cx. pipiens* and *Cx. quinquefasciatus* are the main vectors involved in WNV transmission across Africa and the Americas [5–9].

In Argentina, WNV was first detected in 2006 during an equine encephalitis outbreak, although its introduction was likely earlier, as seropositive birds were identified in a retrospective study [10,11]. There is limited knowledge about which mosquito species are vectoring WNV in Argentina. To date, vector competence (VC) assays have shown that *Cx. quinquefasciatus* and *Cx. pipiens f. molestus* from La Plata were inefficient at transmitting the NY99 and WN02, WNV strains originally isolated in the United States [12]. On the other hand, *Cx. quinquefasciatus* from Córdoba were efficient at transmitting the local WNV strain [5].

Assessing VC is essential to understanding the specific interaction between a viral strain and a potential vector species or population. Vector competence comprises three key components: *(i)* viral infection of the mosquito’s midgut, *(ii)* viral dissemination to other organs, and *(iii)* transmission resulting from the release of progeny virions into the saliva. These components represent sequential steps of the infection process, encompassing viral replication, dissemination to secondary tissues, and ultimately transmission to a susceptible host. Evaluating each stage separately allows the identification of potential anatomical and physiological barriers that constrain viral replication, dissemination, and transmission within mosquito populations [13,14].

While vector competence describes the biological processes that enable a mosquito to become infectious, virus transmission is also constrained by time. Following ingestion of an infectious bloodmeal, WNV must complete replication and dissemination within the mosquito before transmission becomes possible, a period known as the extrinsic incubation period (EIP) [15]. The duration of the EIP reflects the rate at which these infection processes occur and directly influences transmission dynamics by determining when an infected mosquito becomes capable of transmitting the virus during its lifespan [16,17].

Vector competence can be influenced by factors affecting the interaction between the mosquito and the virus throughout the infection process [18,19]. In particular, genetic variation among mosquito species and populations [20–25], as well as among viral strains [20,26,27], can modify infection, dissemination, and transmission outcomes. Likewise, extrinsic factors such as the incubation temperature and the viral dose have been extensively studied and are known to significantly modulate VC outcomes [19]. These factors not only influence the probability of infection, dissemination, and transmission, but also affect the timing of these processes, thereby modulating the EIP. Both intrinsic and extrinsic factors interact to influence the infection dynamics within the vector and, consequently, its ability to transmit the virus.

In this context, this study aimed to determine the vector competence of *Culex pipiens* complex populations from Argentina for West Nile virus across a range of viral doses, evaluate interspecific and intraspecific variation among mosquito populations, and estimate the EIP in a local *Culex* population to characterize the temporal constraints governing virus transmission.

## Methods

### Ethics statement

This study was approved by the Institutional Committee for the Care and Use of Laboratory Animals (CICUAL) of the Faculty of Medical Sciences, National University of Córdoba, under approval number CE-2022–00518476-UNC-SCT#FCM. The Committee was constituted by Cristina López, Ph.D.; Alejandra Báez, Ph.D.; Eugenia Luque, Ph.D.; Clarisa Lagares, Technician; and Arnaldo Mangeaud, Ph.D. All experimental procedures involving animals were conducted in accordance with current national regulations for the use of animals in research.

### Mosquito collection

**Fig 1:**
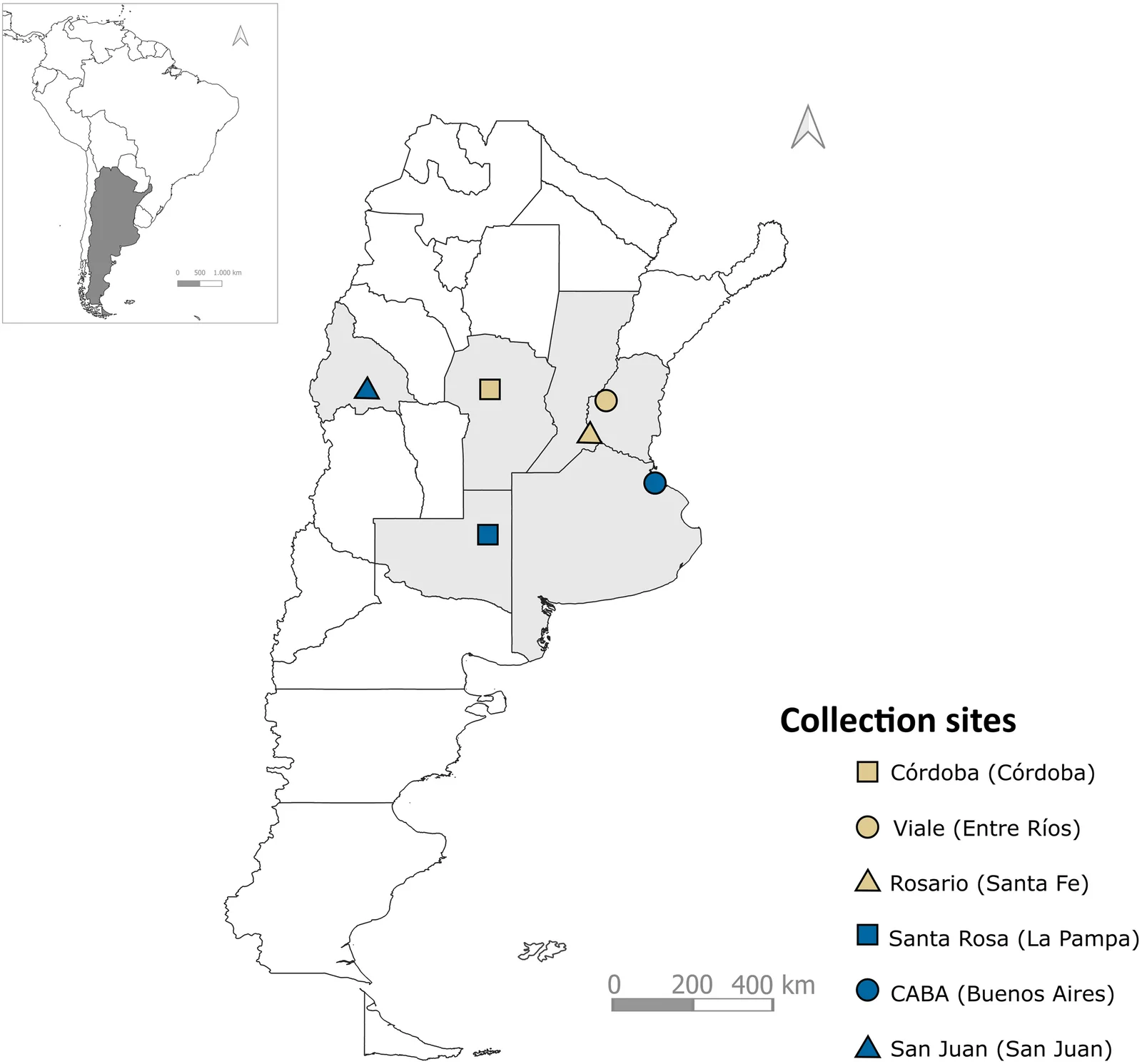
Geographic origin of *Culex* populations used in this study. Map showing the sampling locations of mosquito populations in Argentina. Collection sites are color-coded by species: *Culex pipiens* (blue) and *Culex quinquefasciatus* (yellow). Base map was generated in QGIS using administrative boundary shapefiles obtained from the National Geographic Institute of Argentina (IGN), available at https://www.ign.gob.ar/NuestrasActividades/InformacionGeoespacial/CapasSIG.

Colonies of *Cx. quinquefasciatus* and *Cx. pipiens* were established from egg raft collections conducted during the summer seasons between 2020 and 2024 in different regions of Argentina (Table 1; Fig 1). Field collections were incorporated into the study as they became available, and each mosquito population was maintained as a separate laboratory colony. Experimental infections were conducted independently for each population once colonies had adapted to insectary conditions and reached sufficient numbers to support the infection assays.

**Table 1:** Mosquito populations of *Culex quinquefasciatus* and *Culex pipiens* collected from different provinces of Argentina. The table summarizes collection location, population designation, collection date, and laboratory generation at which experimental infections were performed.

Vector Species	Location (Province)	Population name	Date of collection	Experimental filial generation
*Culex quinquefasciatus*	Córdoba (Córdoba)	Córdoba	01/02/2021	F8
*Culex quinquefasciatus*	Viale (Entre Ríos)	Entre Ríos	01/03/2022	F4
*Culex quinquefasciatus*	Rosario (Santa Fe)	Santa Fe	07/03/2024	F8
*Culex pipiens*	Santa Rosa (La Pampa)	La Pampa	01/03/2020	F20
*Culex pipiens*	CABA (Buenos Aires)	Buenos Aires	20/03/2024	F5
*Culex pipiens*	San Juan (San Juan)	San Juan	15/06/2024	F9

CABA = Ciudad Autónoma de Buenos Aires.

After field collection, individual egg rafts were transferred to the insectary at the Arbovirus Laboratory, Institute of Virology “Dr. J.M. Vanella”, and reared separately through adult emergence. Mosquitoes were maintained under the same controlled environmental conditions: 27°C, 80% relative humidity, and a 12:12 h light:dark photoperiod [28]. Larvae were reared in plastic trays (30 × 20 × 15 cm) containing 1.5 liters of distilled water and were fed powdered liver (0.25 g/day) until pupation. Initial species identification was performed using fourth-instar larvae and adult females from each egg raft, following the morphological keys of Darsie [29], allowing discrimination from other non-target *Culex* species present in the region. Identification within the *Cx. pipiens* complex was further resolved based on male genitalia morphology [29]. Taxonomic identification of *Cx. pipiens* and *Cx. quinquefasciatus* was further confirmed using the PCR developed by Smith & Fonseca (2004). This molecular analysis targets polymorphisms in the second intron of the acetylcholinesterase-2 (*ace-2*) gene, which allows discrimination among members of the *Cx. pipiens* complex and their hybrids [30]. Only egg rafts consistently identified as the target species by both morphological and molecular approaches were incorporated into laboratory colonies, which were subsequently established according to species and collection site (Table 1).

Adult mosquitoes were maintained in 26 × 22 cm cardboard cages and provided with cotton pads soaked in a 10% sucrose solution. All adult colonies were kept under the same environmental conditions used for larval rearing [9,31].

### Viral infection

The WNV strain ArEq001 (GenBank accession no. GQ379160.1) was originally isolated in 2006 from the brain of one of three horses (*Equus caballus*) that died from encephalitis in central Argentina [11]. Phylogenetic analysis classified this strain within clade 1A of lineage I, grouping it with North American strains isolated between 1999 and 2002 [32]. The viral stock was amplified through two passages in *Aedes albopictus* C6/36 cells followed by two passages in Vero cells. For experimental infections, the WNV stock was produced on Vero cells and stored at -80°C until use.

Adult female mosquitoes (5–10 days old) were separated and starved through sugar deprivation 24 hours before the exposure to the infectious bloodmeal. Oral infection was performed using viremic chicks.

All chicks were subcutaneously inoculated at the same time with 100 μl of a WNV suspension. The inoculum was independently titrated for each experiment and ranged from approximately 300–500 plaque forming units (PFU). Mosquitoes were subsequently exposed to viremic chicks at 12, 24, 36, 48, 60, and 72 hours post-inoculation. This approach aimed to generate a range of viremia levels by taking advantage of the natural viral amplification dynamics in each individual host. Chicks were housed in rearing cages with sterile wood shavings, under a 14:10 h light:dark photoperiod at a constant temperature of 25 °C. Food and water were provided *ad libitum*. Euthanasia was performed immediately after mosquito feeding and before blood collection.

One infected chick was offered as an infected blood source for each combination of viral dose and mosquito population, resulting in a total of 36 chicks. After a one-hour feeding period, mosquitoes were anesthetized with CO_2_, and fully engorged females were selected. Immediately after chick removal, 100 μl of blood was collected from the jugular vein to determine the viremia level to which mosquitoes had been exposed. Whole blood was diluted in 0.9 ml of minimum essential medium (MEM) supplemented with 10% fetal bovine serum (FBS) and centrifuged at 1500 g for 15 minutes. Virus titration was performed using 10-fold serial dilutions in Vero 76 cells. Selected females were maintained under the same rearing conditions until euthanasia and sample collection.

### Experimental infection assays and transmission dynamics

A total of 1102 orally infected female mosquitoes were used in this study, distributed across 40 experimental treatments comprising six populations from two species of the *Culex pipiens* complex, exposed to increasing viral doses ranging from 2 log_10_ – 7.5 log_10_ PFU/ml. Experimental procedures followed the standardized guidelines proposed by Wu *et al.,* 2023 for vector competence studies, with full methodological details provided in the S1 Table [33].

Following exposure to an infectious bloodmeal, mosquitoes were maintained under controlled insectary conditions. Vector competence outcomes were assessed at 10 days post-infection (dpi) for all populations. To further analyze transmission dynamics, the *Cx. quinquefasciatus* population from Córdoba exposed to the highest viral dose (7.3 log_10_ PFU/ml), was also assessed and processed at multiple time points (3, 5, 7, 10, and 12 dpi) to evaluate the EIP.

After the incubation period, mosquitoes were anesthetized using triethylamine, and samples were collected separately from the abdomen, legs, and saliva. Saliva was obtained using the capillary tube method [34]. Briefly, the proboscis of each mosquito was inserted into a capillary tube containing 10 μL of MEM (GIBCO) supplemented with 10% FBS (GIBCO) and 10% sucrose and salivation was allowed to proceed for 30 minutes. Abdomen, leg and saliva samples were individually placed into 300 μL of MEM supplemented with 10% FBS. All samples were stored at −80°C until further processing. The presence of infectious viral particles was determined for each sample by plaque assay on Vero cell monolayers, following standard protocols [35,36].

For each mosquito, infective status was determined based on the anatomical distribution of virus infection (abdomen, legs, saliva). A mosquito was considered infected when replicating virus was detected in the abdomen. If virus was detected in the abdomen but not in the legs, the infection was considered restricted to the midgut. Disseminated infection was defined by the presence of replicating virus in both the abdomen and legs, indicating that the virus had successfully overcome the midgut and spread through the hemocoel. Mosquitoes were considered competent for transmission when infectious virus was detected in the saliva. The presence of viral particles in the saliva was interpreted as evidence of successful infection of the salivary glands and the ability to transmit the virus during subsequent blood feeding.

### Statistical analysis

To evaluate VC across viral doses and to assess both interspecific and intraspecific variability, a series of generalized linear mixed models (GLMMs) with a binomial error distribution and a logit link function were fitted. Three binary response variables were analyzed independently for each mosquito: infection, dissemination, and transmission, coded as 0 (negative) or 1 (positive) for viral particle presence in midgut, legs and saliva, respectively. Dissemination and transmission outcomes were modeled using all exposed mosquitoes, rather than conditioning analyses on prior infection or dissemination status. Viral dose (log_10_ transformed) was included as a continuous predictor in all models.

To compare VC between *Culex pipiens* and *Culex quinquefasciatus*, we fitted three GLMMs, one for each response variable, including viral dose, species, and their interaction as fixed effects. Population nested within species was incorporated as a random intercept to account for non-independence among mosquitoes originating from the same population. Initially, we evaluated a more complex random-effects structure that included random slopes for viral dose. However, these models resulted in singular fits, indicating that the variance associated with random slopes was negligible. The significance of fixed effects was assessed using parametric bootstrap with 1000 simulations. Model diagnostics and convergence were evaluated following standard procedures [37].

To evaluate transmission dynamics and estimate the EIP, two generalized linear models (GLMs) with a binomial error distribution and logit link function were fitted using mosquito infection and transmission status as response variables and time (i.e., dpi) as the explanatory variable, using data from the *Cx. quinquefasciatus* Córdoba population sampled at five timepoints (3, 5, 7, 10, and 12 dpi) under the highest viral dose. Due to methodological constraints, experimental observations could not be extended beyond the latest sampling time point, and the temporal plateau in transmission probability was therefore not directly observed.

All regression curves and associated confidence intervals were generated from fitted models. Model-based predicted probabilities were used both for visualization and for the estimation of biologically meaningful thresholds for infection, dissemination, and transmission. Specifically, thresholds were defined as the values of the explanatory variable at which the predicted probability of the outcome equaled 0.5. Accordingly, we estimated the doses at which the predicted probability of becoming infected (ID_50_), disseminating (DD_50_), and transmitting the virus (TD_50_) reached 0.5. Using the same approach, EIP thresholds were defined as the time points at which the predicted probability of infection or transmission reached 0.50 (infection and transmission EIP_50_).

Intraspecific variability was assessed through the random-effects structure of the mixed models, allowing baseline differences among populations to be captured while accounting for the fixed effects of viral dose and species. The magnitude of population-level heterogeneity was quantified using variance components and intraclass correlation coefficients (ICCs).

All statistical analyses were performed in R [38]. Mixed models were fitted using *lme4* package and the significance was evaluated using parametric bootstrap procedures implemented in the *afex* package. Model predictions and confidence intervals were obtained using the *ggeffects* package. Predicted values were subsequently visualized using *ggplot2*. Random-effect structures were inspected and visualized using functions from *lattice*, *sjPlot*, and base *lme4*. We employed *DHARMa* and *broom.mixed* packages to assess model assumptions. Fig 1 was created in QGIS (version 4.0; QGIS Association) using official administrative boundary shapefiles obtained from the National Geographic Institute of Argentina (Instituto Geográfico Nacional, IGN) through its public geospatial database. No proprietary cartographic products or commercial basemap services were used in the preparation of this figure. Raw data and analysis code are available at https://doi.org/10.6084/m9.figshare.33049649.

## Results

PCR-based identification yielded 22, 17, and 29 egg rafts consistent with *Cx. quinquefasciatus* (274 bp) for the Córdoba, Entre Ríos, and Santa Fe populations, respectively, and 15, 19, and 16 egg rafts consistent with *Cx. pipiens* (610 bp) for the La Pampa, Buenos Aires, and San Juan populations, respectively; no double-band (610 + 274 bp) hybrid-diagnostic pattern was detected in any of the six colonies.

**Fig 2:**
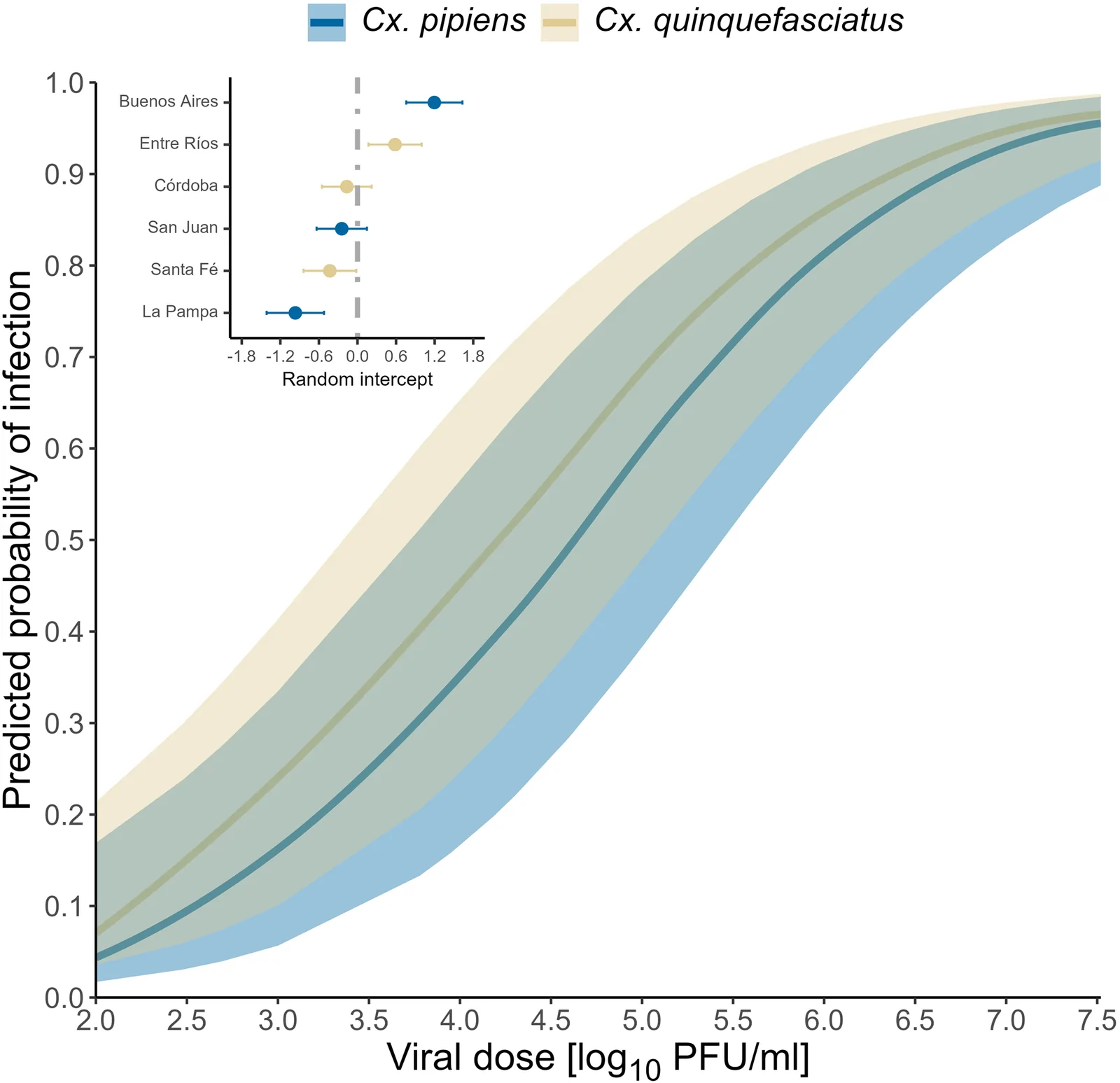
Dose–response relationship for West Nile virus infection in two species of the *Culex pipiens* complex. Predicted probability of infection is shown as a function of viral dose (log₁₀ PFU/ml) for *Cx. pipiens* (blue) and *Cx. quinquefasciatus* (yellow). Solid lines represent model-predicted probabilities, and shaded areas indicate 95% model-based confidence intervals. Embedded inset shows population-level random intercept estimates on the logit scale (± 2 standard errors), illustrating population-specific variability around the global mean response.

**Fig 3:**
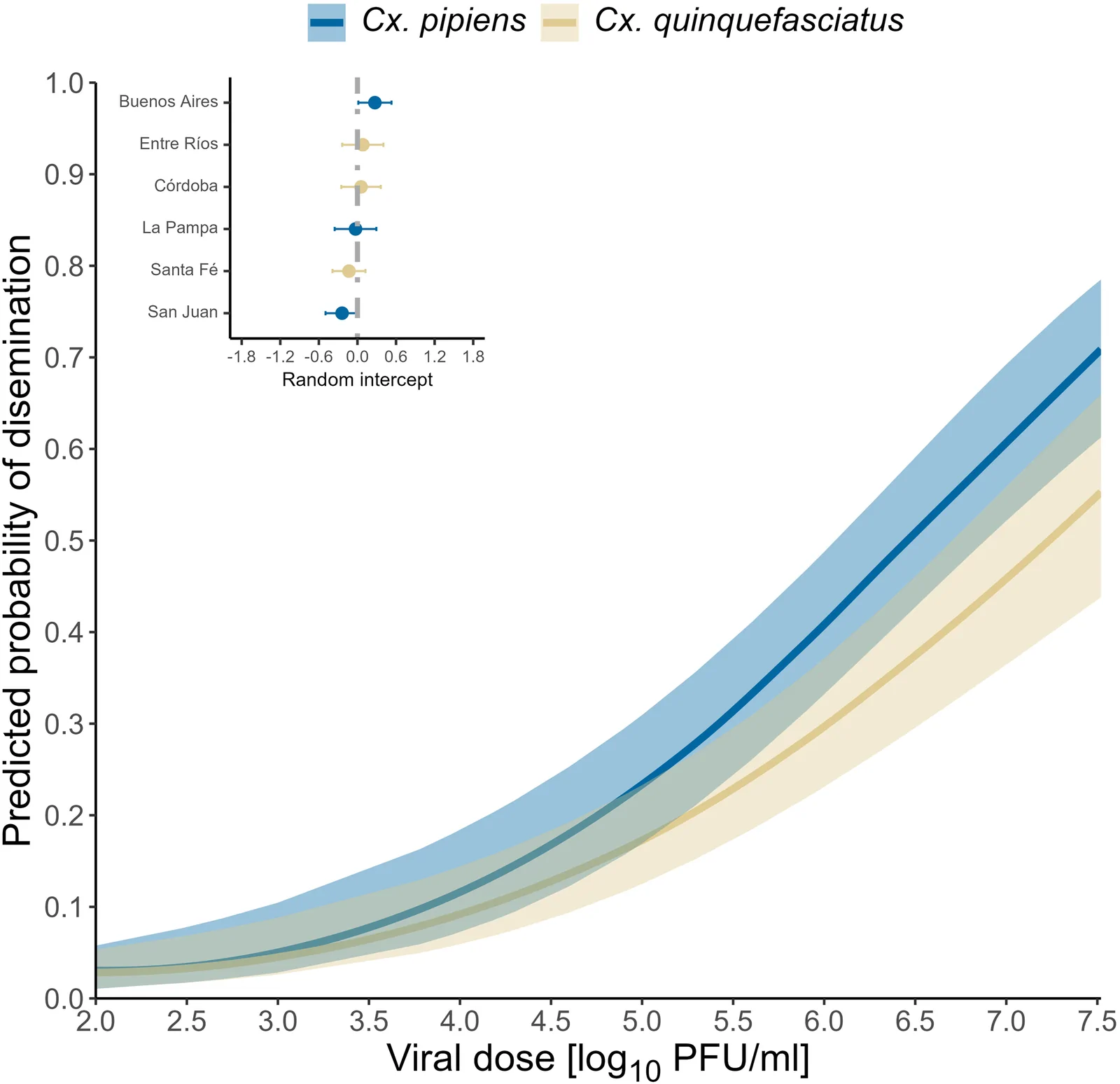
Dose–response relationship for West Nile virus dissemination in two species of the *Culex pipiens* complex. Predicted probability of dissemination is shown as a function of viral dose (log₁₀ PFU/ml) for *Cx. pipiens* (blue) and *Cx. quinquefasciatus* (yellow). Solid lines represent model-predicted probabilities, and shaded areas indicate 95% model-based confidence intervals. Embedded inset shows population-level random intercept estimates on the logit scale (± 2 standard errors), illustrating population-specific variability around the global mean response.

**Fig 4:**
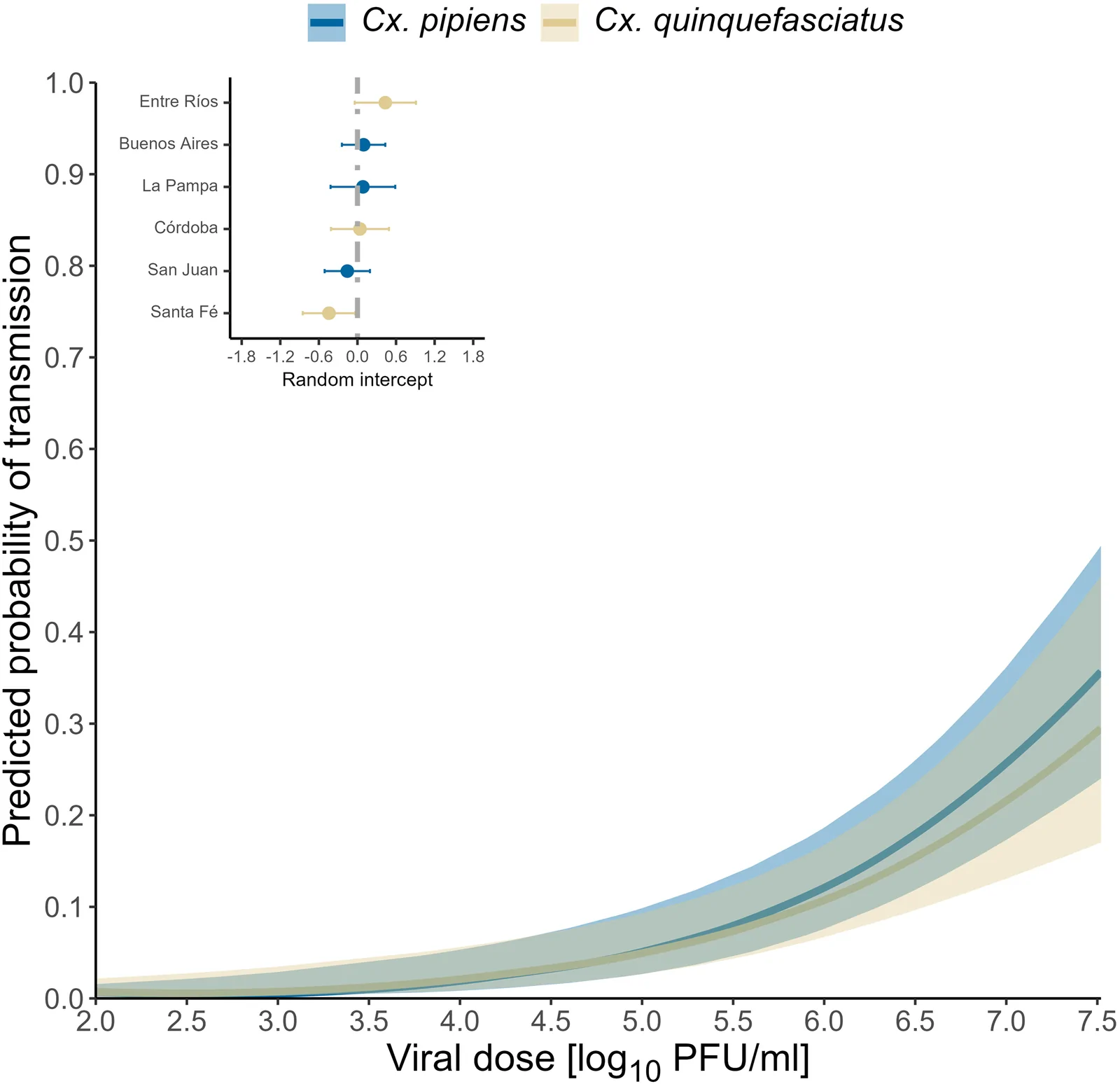
Dose–response relationship for West Nile virus transmission in two species of the *Culex pipiens* complex. Predicted probability of transmission is shown as a function of viral dose (log₁₀ PFU/ml) for *Cx. pipiens* (blue) and *Cx. quinquefasciatus* (yellow). Solid lines represent model-predicted probabilities, and shaded areas indicate 95% model-based confidence intervals. Embedded inset shows population-level random intercept estimates on the logit scale (± 2 standard errors), illustrating population-specific variability around the global mean response.

To determine whether VC differed between species and across levels of host viremia, we evaluated the effects of viral dose and mosquito species on infection, dissemination, and transmission probabilities. Generalized linear mixed models revealed a consistent and strong positive effect of viral dose on the probability of mosquito infection (Fig 2), dissemination (Fig 3) and transmission (Fig 4) (infection: χ² = 265.60, df = 1, p < 0.001; dissemination: χ² = 171.09, df = 1, p < 0.001; transmission: χ² = 85.91, df = 1, p < 0.001). In contrast, no significant differences were detected between species for any of the three outcomes (infection: χ² = 0.36, p = 0.567; dissemination: χ² = 0.06, p = 0.796; transmission: χ² = 0.08, p = 0.781), nor were there significant dose × species interactions (all p > 0.43), indicating parallel dose–response relationships in *Cx. pipiens* and *Cx. quinquefasciatus*. These results indicate that increasing viremia enhanced infection, dissemination, and transmission in both species, while overall vector competence responses remained broadly similar between *Cx. pipiens* and *Cx. quinquefasciatus* (raw data summarized in Table 2).

**Table 2:** Raw infection, dissemination, and transmission data for each *Culex* population at each viremia evaluated. For each species, population, and viral dose, the table reports the number of mosquitoes exposed, and the infection, dissemination, and transmission rates.

Species	Population	Viral dose (log_10_ PFU/ml)	Infection rate (n° infected/ tested)	Dissemination rate (n° disseminated/ tested)	Transmission rate (n° transmitted/ tested)	Days post inoculation (DPI)
*Cx. quinquefasciatus*	Córdoba	2.00	0.13 (4/31)	0 (0/31)	0 (0/31)	10
*Cx. quinquefasciatus*	Córdoba	3.00	0.22 (7/32)	0 (0/32)	0 (0/32)	10
*Cx. quinquefasciatus*	Córdoba	4.30	0.44 (15/34)	0.18 (6/34)	0 (0/34)	10
*Cx. quinquefasciatus*	Córdoba	4.60	0.48 (12/25)	0.24 (6/25)	0.08 (2/25)	10
*Cx. quinquefasciatus*	Córdoba	6.85	0.91 (29/32)	0.31 (10/32)	0.09 (3/32)	10
Cx. quinquefasciatus*	Córdoba*	7.30*	1 (18/18)*	0.72 (13/18)*	0.5 (9/18)*	10*
Cx. quinquefasciatus*	Córdoba*	7.30*	0.28 (7/25)*	0.16 (4/25)*	0 (0/25)*	3*
Cx. quinquefasciatus*	Córdoba*	7.30*	0.76 (16/21)*	0.29 (6/21)*	0 (0/21)*	5*
Cx. quinquefasciatus*	Córdoba*	7.30*	0.91 (20/22)*	0.32 (7/22)*	0 (0/22)*	7*
Cx. quinquefasciatus*	Córdoba*	7.30*	0.84 (26/31)*	0.65 (20/31)*	0.58 (18/31)*	12*
*Cx. quinquefasciatus*	Entre Ríos	2.48	0.06 (2/32)	0 (0/32)	0 (0/32)	10
*Cx. quinquefasciatus*	Entre Ríos	3.95	0.8 (28/35)	0.17 (6/35)	0.03 (1/35)	10
*Cx. quinquefasciatus*	Entre Ríos	5.30	0.86 (30/35)	0.26 (9/35)	0.2 (7/35)	10
*Cx. quinquefasciatus*	Entre Ríos	6.00	0.86 (30/35)	0.29 (10/35)	0.17 (6/35)	10
*Cx. quinquefasciatus*	Santa Fe	2.70	0.07 (2/30)	0 (0/30)	0 (0/30)	10
*Cx. quinquefasciatus*	Santa Fe	5.28	0.73 (11/15)	0.2 (3/15)	0 (0/15)	10
*Cx. quinquefasciatus*	Santa Fe	6.28	0.91 (20/22)	0.18 (4/22)	0 (0/22)	10
*Cx. quinquefasciatus*	Santa Fe	6.30	0.87 (26/30)	0.4 (12/30)	0.13 (4/30)	10
*Cx. quinquefasciatus*	Santa Fe	6.60	0.9 (27/30)	0.37 (11/30)	0.13 (4/30)	10
*Cx. quinquefasciatus*	Santa Fe	6.78	0.93 (25/27)	0.44 (12/27)	-	10
*Cx. quinquefasciatus*	Santa Fe	7.00	0.84 (16/19)	0.42 (8/19)	0.16 (3/19)	10
*Cx. quinquefasciatus*	Santa Fe	7.48	0.87 (26/30)	0.4 (12/30)	0.1 (3/30)	10
*Cx. pipiens*	La Pampa	2.00	0 (0/21)	0 (0/21)	0 (0/21)	10
*Cx. pipiens*	La Pampa	4.90	0.23 (5/22)	0.05 (1/22)	0 (0/22)	10
*Cx. pipiens*	La Pampa	5.00	0.25 (5/20)	0.1 (2/20)	0 (0/20)	10
*Cx. pipiens*	La Pampa	6.00	0.72 (13/18)	0.56 (10/18)	0.22 (4/18)	10
*Cx. pipiens*	La Pampa	6.41	0.8 (16/20)	0.65 (13/20)	0.25 (5/20)	10
*Cx. pipiens*	Buenos Aires	3.78	0.63 (19/30)	0.17 (5/30)	0.07 (2/30)	10
*Cx. pipiens*	Buenos Aires	4.18	0.7 (21/30)	0.3 (9/30)	0.07 (2/30)	10
*Cx. pipiens*	Buenos Aires	5.90	0.93 (28/30)	0.47 (14/30)	0.07 (2/30)	10
*Cx. pipiens*	Buenos Aires	6.30	0.93 (28/30)	0.53 (16/30)	0.13 (4/30)	10
*Cx. pipiens*	Buenos Aires	6.65	1 (30/30)	0.7 (21/30)	0.27 (8/30)	10
*Cx. pipiens*	Buenos Aires	7.46	0.93 (28/30)	0.57 (17/30)	0.3 (9/30)	10
*Cx. pipiens*	Buenos Aires	7.52	1 (30/30)	0.73 (22/30)	0.37 (11/30)	10
*Cx. pipiens*	San Juan	4.90	0.57 (17/30)	0.03 (1/30)	0 (0/30)	10
*Cx. pipiens*	San Juan	5.60	0.67 (20/30)	0.4 (12/30)	0.1 (3/30)	10
*Cx. pipiens*	San Juan	6.48	0.72 (18/25)	0.08 (2/25)	0.04 (1/25)	10
*Cx. pipiens*	San Juan	6.85	0.9 (27/30)	0.43 (13/30)	0.17 (5/30)	10
*Cx. pipiens*	San Juan	7.00	0.93 (26/28)	0.68 (19/28)	0.18 (5/28)	10
*Cx. pipiens*	San Juan	7.40	0.97 (36/37)	0.73 (27/37)	0.41 (15/37)	10

* Mosquitoes from the *Cx. quinquefasciatus* Córdoba population, exposed to the highest viral dose and sampled at 3, 5, 7, 10, and 12 days post-infection (dpi) for the temporal characterization and estimation of the extrinsic incubation period (EIP).

To quantify the magnitude of the dose effect, we examined model-derived effect sizes for each stage of VC. Effect size estimates showed that each 1-log10 increase in viral dose (equivalent to a tenfold increase in viremia) increased the odds of infection by a factor of 2.93 (β = 1.0738 ± 0.1328 SE, Fig 2), dissemination by 2.28 (β = 0.8228 ± 0.0994 SE, Fig 3), and transmission by 2.51 (β = 0.9199 ± 0.1616 SE, Fig 4), corresponding to increases of approximately 193%, 128%, and 151% in odds, respectively.

To characterize the viral doses required for successful infection and onward transmission, we estimated dose thresholds from the fitted models. Because neither species nor species-by-dose interactions were significant, threshold estimates were comparable between *Cx. pipiens* and *Cx. quinquefasciatus*. For infection, ID_50_ was reached at 4.63 log₁₀ PFU/ml (Fig 2). Dissemination required higher doses, with a DD_50_ at 6.45 log₁₀ PFU/ml (Fig 3). Transmission exhibited the highest thresholds, with TD_50_ at 8.16 log₁₀ PFU/ml (Fig 4). Because the highest experimentally observed viremia was approximately 7.5 log₁₀ PFU/ml, this value was obtained by extrapolating the fitted logistic regression beyond the range of observed data (i.e., taking the x-axis value of the obtained logistic regression function at which the predicted probability equals 0.5), and should therefore be interpreted with caution. These results suggest that progressively higher host viremias are required for mosquitoes to overcome successive barriers to infection, dissemination, and ultimately transmission under the designed experimental conditions.

Because species-level responses were largely homogeneous, we next explored whether vector competence varied among populations within each species. Intraspecific variability was assessed by examining the random intercepts associated with population nested within species. For infection, population-level heterogeneity was substantial (ICC = 14.1%), and the conditional modes of the random effects revealed marked deviations from the overall mean response. Within *Cx. pipiens*, the population from Buenos Aires showed a strong positive deviation in baseline infection probability, whereas La Pampa exhibited a negative deviation. Among *Cx. quinquefasciatus* populations, Entre Ríos showed a positive deviation, while Santa Fe was below the overall mean (Fig 2, embedded inset). In contrast, population-level variability was minimal for dissemination (ICC = 1.4%), with all populations showing small deviations around the model mean, indicating a largely homogeneous dissemination response once infection was established (Fig 3, embedded inset). For transmission, population-level variance remained also low (ICC = 3.4%), but notable deviations were observed in specific *Cx. quinquefasciatus* populations: Entre Ríos showed a positive deviation in baseline transmission probability, whereas Santa Fe exhibited a negative deviation (Fig 4, embedded inset). Overall, population-level differences were most evident during the infection stage, whereas dissemination and transmission responses were comparatively uniform once infection had been established.

**Fig 5:**
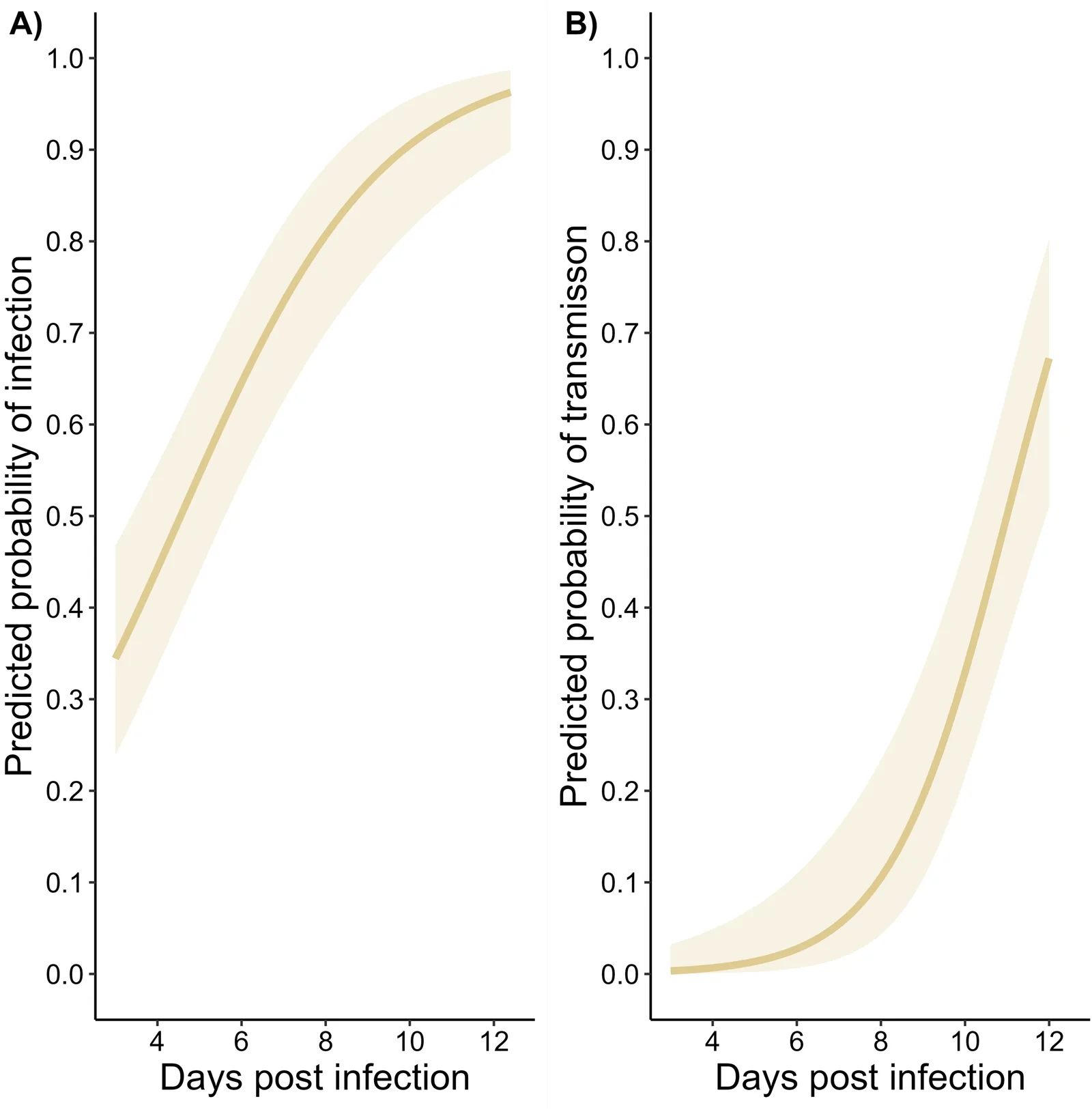
Temporal dynamics of West Nile virus infection and transmission in *Culex quinquefasciatus.* Predicted probabilities are shown as a function of days post-infection (dpi) for mosquitoes exposed to the highest viral dose. **(A)** Probability of infection over time. **(B)** Probability of transmission over time. Solid lines represent model-predicted probabilities, and shaded area indicate 95% model-based confidence intervals.

To characterize the temporal dynamics of WNV infection and transmission, and to estimate the extrinsic incubation period (EIP), we evaluated how infection and transmission probabilities changed over time in *Cx. quinquefasciatus* mosquitoes exposed to the highest viral dose (Table 2). Binomial GLMs revealed a strong positive effect of time (i.e., dpi) on both infection and transmission in *Cx. quinquefasciatus* exposed to the highest viral dose. Infection probability increased significantly with dpi (β = 0.415 ± 0.069 SE, z = 6.03, p < 0.001), corresponding to a 51% increase in the odds of infection per additional day post-infection, with model-based predictions indicating an estimated infection EIP₅₀ of approximately 4.6 dpi (Fig 5A). Transmission probability also increased significantly over time (β = 0.715 ± 0.151 SE, z = 4.75, p < 0.001), reflecting an approximately twofold increase in the odds of transmission per additional day. Model predictions indicated that the onset of transmission occurred at approximately 7.9 dpi, whereas the transmission EIP₅₀ was reached at approximately 11.0 dpi (Fig 5B). These results indicate that WNV infection became established rapidly following exposure, whereas additional time was required for viral dissemination to the salivary glands and the subsequent acquisition of transmission competence.

## Discussion

A detailed understanding of how virus-vector interactions are structured in local *Culex* species is essential for improving knowledge of WNV ecoepidemiology at its southern limit of distribution. In this study, dose–response relationships were quantitatively characterized across the sequential processes of VC (infection, dissemination, and transmission) in two members of the *Culex pipiens* complex while integrating the temporal dimension of infection dynamics. Experimental infections using multiple viral doses across populations from Argentina allowed the evaluation of how *Cx. pipiens* and *Cx. quinquefasciatus* respond to increasing viral doses while explicitly evaluating population-level variation within each species. Both species exhibited broadly overlapping dose–response patterns and comparable infectious and transmission dose thresholds. Despite these similarities, intraspecific variability emerged as a prominent feature of the response, particularly during the initial infection stage, with certain populations showing increased susceptibility.

This study did not detect interspecific differences in the dose–response relationship between *Cx. pipiens* and *Cx. quinquefasciatus* across the evaluated components of vector competence. Under the highest viremia evaluated (7.5 log_10_ PFU/ml), both species exhibited predicted transmission probabilities close to 30%, although population-level estimates varied from approximately 10% to 50%. In Europe, vector competence of *Cx. pipiens* has been extensively characterized, with a comprehensive review integrating multiple experimental studies reporting heterogeneous transmission outcomes across geographically distinct populations, with maximum transmission rates generally ranging between approximately 30% and 60% [6]. This range has been consistently supported by subsequent experimental studies in northern and western Europe, including populations from France, Finland, and Belgium, which have confirmed the ability of *Cx. pipiens* to transmit WNV under controlled laboratory conditions while maintaining substantial variability in transmission efficiency across populations [39–41]. In the United States, a review of vector competence studies has highlighted substantial heterogeneity in transmission outcomes for both *Cx. pipiens* and *Cx. quinquefasciatus*, with transmission levels generally averaging around 20% but reaching up to approximately 50% under higher viral doses [42]. Illustrating this variability, Kilpatrick et al. reported that *Cx. pipiens* populations from different regions of the United States exhibited transmission rates ranging from 0% to 52% following exposure to a bloodmeal containing 7 log₁₀ PFU/ml, emphasizing that vector competence varies markedly across geographic regions and temporal scales. Likewise, Goddard et al. evaluated both *Cx. pipiens* and *Cx. quinquefasciatus* under comparable experimental conditions (7 log₁₀ PFU/ml; 14 dpi), reporting transmission rates ranging from 19% to 52% for *Cx. quinquefasciatus* and up to 71% for *Cx. pipiens*. In South America, where data remains scarce, studies in Brazil and Argentina have reported high infection rates at high doses, with dissemination and transmission remaining comparatively lower [5,12,43]. Particularly for Argentina, Micieli et al. reported low or absent transmission in *Cx. pipiens* f. *molestus* (8.8% and 0%) and *Cx. quinquefasciatus* (16.7% and 9.1%) following oral exposure to the North American WNV genotypes NY99 and WN02 at viral doses between 7.8 and 8.5 log_10_ PFU/ml [12]. These results contrast markedly with those obtained here and in our previous work [5], where transmission was detected at substantially higher rates using the Argentine strain ArEq001. Although differences in mosquito population origin may contribute, the use of distinct viral strains is the most parsimonious explanation for this discrepancy. Notably, despite sharing approximately 99.5% amino acid identity with NY99, ArEq001 may differ in phenotypic traits affecting mosquito infection and transmission, although the relative contribution of viral and mosquito genetic backgrounds cannot be disentangled from the available data. Overall, direct quantitative comparisons among vector competence studies should be interpreted cautiously because experimental protocols differ substantially in viral strains, infectious doses, feeding methods, incubation temperatures, and sampling schedules. Despite these methodological differences, the overall dose–response patterns observed here are broadly consistent with those reported for members of the *Culex pipiens* complex evaluated under comparable experimental conditions, particularly when mosquito populations are challenged with geographically relevant WNV strains.

From a dose–response perspective, barriers to infection, dissemination, and transmission can be interpreted as processes that shape both the position and the form of the response curve. The position of the curve, reflected by ~D_50_ values (i.e., ID_50_, DD_50_ and TD_50_ for infection, dissemination and transmission probabilities respectively), describes the viral dose required to achieve a given probability of the outcome, whereas the slope describes how rapidly that probability increases once the threshold is exceeded. In our study, successive stages of the infection process were characterized by progressively higher ~D_50_ values, indicating that increasingly greater host viremias were required to achieve comparable probabilities of infection, dissemination, and transmission within a common incubation period. At the same time, the midgut infection barrier exhibited the steepest dose–response slope, indicating that relatively small increases in dose beyond the threshold resulted in rapid increases in infection probability. In contrast, dissemination and transmission displayed progressively shallower slopes, suggesting that constraints acting at later stages of infection are overcome more gradually across the range of viral doses evaluated. Together, these patterns suggest that vector competence barriers differ not only in their threshold location but also in the rate at which probabilities increase once those thresholds are surpassed. These differences in how barriers are defined and overcome likely reflect the multifactorial nature of vector competence [44], not only involving anatomical filters [45], but also immunological, physiological, and microbiota-related processes [46–48]. Here, a similar pattern of putative barriers was observed in both species, evidenced by the lack of a significant dose and species interaction and their shared response relative to the global mean fixed-effects of the model.

The substantial population-level heterogeneity observed during the infection stage suggests that processes associated with the midgut infection barrier may represent an important source of variation in vector competence. Because the present study was not designed to investigate the mechanisms underlying these differences, the factors responsible for this variability remain unresolved. Previous studies have shown that mosquito susceptibility to WNV may be influenced by population-specific immune responses, physiological trade-offs, and microbiota composition [48–54]. Future studies integrating vector competence assays with transcriptomic, physiological, and microbiome analyses will be necessary to identify the mechanisms underlying the population-level differences observed here. Population-level variance was substantially lower for dissemination and transmission than for infection. Although this pattern suggests relatively homogeneous responses once infection was established, the lower number of positive dissemination and transmission events may also have reduced the statistical power to detect subtle population-specific differences at these later stages.

A temporal lag of approximately 4–7 days was observed between the establishment of infection and the onset of transmission, consistent with tissue tropism dynamics previously described for WNV, in which viral dissemination progresses sequentially from the midgut epithelium to secondary tissues and ultimately the salivary glands [55]. The estimated values for the onset (8 dpi) and median (11 dpi) EIP observed here fall within the range reported for North American populations of *Culex quinquefasciatus* maintained under comparable experimental conditions, where transmission typically begins between 8 and 12 dpi [19,55,56]. Despite agreement with previous observations, modelling transmission as a function of time suggests that vector competence cannot be fully interpreted through viral dose alone. Instead, transmission probability emerges from the interaction between dose, environmental temperature and incubation time, indicating that temporal progression plays a central role once infection has been established. Consistent with earlier hypotheses proposing that vector competence dynamics become predominantly time-dependent after the midgut escape barrier is overcome [19], our results support a shift from dose-limited to time-limited processes during later stages of infection. However, most experimental studies have evaluated the effect of dose, temperature and time separately, preventing a quantitative characterization of their joint effects. Integrating these variables within unified regression frameworks will therefore be essential to describe the dose–time–temperature response surface governing transmission, ultimately improving eco-epidemiological predictions under changing climatic conditions.

The present study provides evidence that *Cx. pipiens* and *Cx. quinquefasciatus* exhibit comparable levels of vector competence for WNV under controlled laboratory conditions across populations sampled within central Argentina, near the southern limit of the virus distribution. The dose–response relationships, transmission probabilities, and extrinsic incubation period estimated here broadly overlap with those reported for competent populations of the *Cx. pipiens* complex in North America when evaluated under comparable experimental conditions, suggesting that Argentine populations are likely to be similarly competent as their northern counterparts. By explicitly accounting for intraspecific variability, these findings support the interpretation that both species may contribute similarly to local transmission dynamics rather than representing functionally distinct vectors. This epidemiological potential is further reinforced by the ecological context of the region, where both species are highly abundant in urban and peri-urban environments [57,58], have been found naturally infected with WNV [59], and feed on avian hosts commonly implicated in transmission networks [60]. Nonetheless, because species identification relied on a single diagnostic locus (*ace-2*) and gene flow between *Cx. pipiens* and *Cx. quinquefasciatus* in central Argentina remains only partially characterized [58,61], low-level introgression between the two taxa cannot be entirely excluded and could, in principle, have contributed to the similarity observed here, as hybridization within the complex has been shown to alter WNV transmission phenotypes elsewhere [62].

Despite these similarities in intrinsic vector competence, the epidemiology of WNV differs markedly between North and South America. Since its introduction into the Americas in 1999, WNV has become firmly established throughout the United States, causing approximately 63,000 reported human cases, including more than 33,000 cases of neuroinvasive disease, and resulting in recurrent seasonal amplification with frequent spillover [63]. In contrast, South America has experienced comparatively limited viral activity, characterized by sporadic detections in birds, horses, and mosquitoes despite serological evidence of widespread circulation [64]. Our findings, restricted to Cx. pipiens and Cx. quinquefasciatus, suggest that these contrasting epidemiological patterns are unlikely to be explained solely by differences in competence between these two local *Culex* populations. Instead, they might instead be driven by broader ecological processes, including differences in host community composition, vector–host interactions, and enzootic transmission networks. However, the potential contribution of other local *Culex* species to WNV maintenance networks in Argentina remains largely unevaluated. Species such as *Cx. interfor* and *Cx. saltanensis* have been shown to be susceptible and competent vectors for St. Louis encephalitis virus, a related flavivirus that co-circulates in the region [31], but their vector competence and role in WNV transmission dynamics have not yet been characterized. Consequently, comprehensive ecological studies characterizing WNV maintenance and amplification networks in South America are urgently needed. Elucidating these networks will not only advance our fundamental understanding of WNV transmission dynamics but also clarify how land-use modifications influence viral spillover and impact public health [54].

## Supporting Information

S1 Table Dataset supporting vector competence analyses. Experimental data including viral dose, infection, dissemination, and transmission outcomes for each tested mosquito population.
(XLSX)
